# A Multiple-Array SPRi Biosensor as a Tool for Detection of Gynecological–Oncological Diseases

**DOI:** 10.3390/bios13020279

**Published:** 2023-02-16

**Authors:** Beata Szymanska, Zenon Lukaszewski, Kinga Hermanowicz-Szamatowicz, Ewa Gorodkiewicz

**Affiliations:** 1 Faculty of Chemistry, Bioanalysis Laboratory, University of Bialystok, Ciolkowskiego 1K, 15-245 Bialystok, Poland; 2 Faculty of Chemical Technology, Poznan University of Technology, Pl. Sklodowskiej-Curie 5, 60-965 Poznan, Poland; 3 Department of Clinical Oncology, Comprehensive Cancer Center, Ogrodowa 12, 15-027 Bialystok, Poland

**Keywords:** CA125, HE4, CEA, IL-6, aromatase, ovarian cancer, endometriosis

## Abstract

Diagnostics based on the determination of biomarkers in body fluids will be more successful when several biomarkers are determined. A multiple-array SPRi biosensor for the simultaneous determination of CA125, HE4, CEA, IL-6 and aromatase has been developed. Five individual biosensors were placed on the same chip. Each of them consisted of a suitable antibody covalently immobilized onto a gold chip surface via a cysteamine linker by means of the NHS/EDC protocol. The biosensor for IL-6 works in the pg mL^−1^ range, that for CA125 in the µg mL^−1^ range, and the other three within the ng mL^−1^ range; these are ranges suitable for the determination of biomarkers in real samples. The results obtained with the multiple-array biosensor are very similar to those obtained with a single biosensor. The applicability of the multiple biosensor was demonstrated using several examples of plasma from patients suffering from ovarian cancer and endometrial cyst. The average precision was 3.4% for the determination of CA125, 3.5% for HE4, 5.0% for CEA and IL-6, and 7.6% for aromatase. The simultaneous determination of several biomarkers may be an excellent tool for the screening of the population for earlier detection of diseases.

## 1. Introduction

Diagnostics based on the determination of biomarkers in body fluids, such as blood serum/plasma, urine, saliva, cerebro-spinal fluid, etc., are leading tendencies in bioanalytical chemistry. So-called ‘liquid biopsy’ can be expected to reduce the need for a real biopsy. However, progress in developing methods for biomarker determination is slower than was expected, and the pace of their introduction in diagnostics is even slower [1]. The case of the detection of ovarian cancer is a typical example. The determination of characteristic biomarkers, such as CA125 and HE4, in blood serum/plasma is a useful tool for the detection of this cancer. However, a diagnosis based on the results for a single biomarker is not 100% reliable. In the case of ovarian cancer, better diagnostic results are given by the algorithm called ROMA, calculated on the basis of two biomarkers: CA125 and HE4. Unfortunately, the available set of biomarkers is limited and should be enlarged. It seems that the simultaneous determination of several biomarkers using a multiple biosensor may be a promising opportunity to improve on the results obtained with a single biomarker. Such an opportunity is provided by the array SPRi (surface plasmon resonance imaging) technique. To date, no multiple SPR or SPRi biosensors have been developed and used in diagnostics, although diagnostics based on the determination of several biomarkers will be more successful than those based on the determination of a single biomarker.

The array SPRi technique has been developed for the determination of molecular biomarkers in body fluids, and, unlike fluidic SPR, is able to determine molecular biomarkers at necessary levels of concentration without signal enhancement or preliminary preconcentration. The most significant difference from the fluidic version of SPR is that the SPRi measurement is performed after the removal of the processing solution. The array SPRi technique uses a chip consisting of an array of measuring points. Several measuring cells are created from the measuring points using hydrophobic paint. Each cell contains 12 measuring points. Chips containing 4, 6 or 9 measuring cells are used. To improve the precision of measurement, an average of the results from a measuring cell is considered as a single result. Depending on the chip’s architecture, 4, 6 or 9 measurements can be performed simultaneously. To date, multiple measuring cells have been used for the simultaneous determination of the same biomarker in several samples. The aim of the present work is to investigate the possibility of forming a multiple biosensor for the determination of several biomarkers on a single chip using the same chip architecture.

Five biomarkers were selected for determination with a multiple biosensor: CA125, HE4, CEA, IL-6 and aromatase. CA125 and HE4 are known ovarian cancer biomarkers. CEA is principally a colon cancer marker, but its concentration in blood serum/plasma is elevated in the case of numerous other cancers. IL-6 is generally an inflammation biomarker; however, elevated serous IL-6 is also observed in cancers. Aromatase is an emerging biomarker for bladder cancer, ovarian cancer, as well as endometriosis. Methods for the determination of these biomarkers with single purpose biosensors have been developed and reported [2,3,4,5,6].

CA125 (carcinoma antigen 125), also known as mucin 16, is a glycoprotein with hydrophilic and lubricating properties, consisting of over 22,000 amino acids [7]. The masses of different isoforms range from about 190 kDa to 2700 kDa [8]. Apart from the female reproductive tract, CA125 occurs in the epithelia of the respiratory tract and in the ocular surface [7]. CA125 is a biomarker of ovarian cancer [9] and endometrial cancer [10]. Serous biomarker concentration was expressed in activity units (U mL^−1^) or standard concentration units (µg mL^−1^), and was reported to range between 5.4 and 6700 U mL^−1^ for ovarian cancer patients [11], and between 4.9 and 88.5 U mL^−1^ for healthy persons, with a cut-off value of 35 U mL^−1^. Activity is calculated from the concentration using the equation A = a C, where A is the activity (U mL^−1^), C is the concentration (µg mL^−1^) and ‘a’ is the specific activity, which should be determined for each standard solution.

HE4 (human epididymis protein) is a glycoprotein which may be present in several isoforms with different molecular weights [12,13]. Therefore, its concentration is usually expressed in pM (picomoles/L) in order to avoid ambiguity in the results. It is an ovarian cancer biomarker whose serous concentration strongly depends on the stage of ovarian cancer [14]. In cases of ovarian cancer, serous HE4 concentration is above 150 pM (6.6 ng mL^−1^) and may exceed 850 pM (37 ng mL^−1^) [14]. HE4 levels in the serum of healthy premenstrual women are between 15 and 62 pM (0.66–2.7 ng mL^−1^) [15], and the recommended cut-off value is between 70 (3.1 ng/mL) and 87 pM (3.8 ng/mL) for premenstrual women, and between 112 pM (4.9 ng/mL) and 140 pM (6.2 ng/mL) for postmenstrual women [16,17].

CEA (carcinoembryonic antigen) is a glycoprotein with an MW of 180–200 kDa [18], containing 60% carbohydrates [19,20]. The marker is involved in cell recognition and adhesion mechanisms [21]. CEA is a colon cancer marker [22,23,24]. The main site of CEA metabolism is the liver. However, increased CEA levels have also been observed in several other types of cancer, such as breast cancer [25], pancreatic cancer [26,27] and gastric cancer [28]. The normal concentration of the CEA in serum is below 5 ng mL^−1^ [29].

IL-6 (interleukin-6) is a glycoprotein consisting of 212 amino acids with MWs in the range of 21–30 kDa [30]. The biomarker plays a critical role in the control of inflammation and participates in autoimmune inflammation (rheumatoid arthritis and multiple sclerosis). It participates in cytokine storm formation in the advanced stage of COVID-19 [31]. Serum IL-6 concentration is elevated in several types of cancer [32,33,34,35]. Reported IL-6 serum concentrations for healthy controls range between 1.2 pg mL^−1^ and 20 pg mL^−1^ [32,36,37], and levels are higher in the case of patients with ovarian cancer (41.23 pg mL^−1^) or acute appendicitis (up to 1000 pg mL^−1^).

Human aromatase is a 58 kDa protein that consists of 503 amino-acids [38]. It is an enzymatic complex responsible for the biosynthesis of estrogens from androgens [39]. The increased expression of aromatase is critical in the pathology of such diseases as breast cancer, endometriosis and hypogonadism, while a lack of activity or reduced activity of aromatase may cause reduced functioning of brain neurons and favour the development of such diseases as Alzheimer’s disease or Parkinson’s disease [40]. An abnormal high expression of aromatase has been correlated with ovarian cancer and endometriosis [41]. It is also an emerging biomarker of bladder cancer [42]. Reported aromatase serum concentrations for healthy controls range between 2.59 and 7.74 ng mL^−1^, and levels are significantly higher in the case of patients with bladder cancer (17.4–57.44 ng mL^−1^) [42].

In analyzing the possibility of constructing multiple biosensors for five biomarkers on the same chip, it should be observed that all five of the above-mentioned biosensors have the same linker, cysteamine. This makes the task easier. At least the first step of the formation of the multiple biosensor will be the same for the whole chip. However, the subsequent stages will be different for each of the five sites because different antibodies need to be immobilized. Because each of the five biosensors operates in a different concentration range, different concentrations of antibodies should be applied. Based on previous papers [2,3,4,5,6], the concentration of the rabbit polyclonal anti-CA125 antibody was selected as 200 µg mL^−1^, the concentration of the rabbit polyclonal antibody against HE4 as 20 ng mL^−1^, the concentration of the mouse monoclonal anti-CEA antibody as 80 ng mL^−1^, the concentration of the mouse monoclonal anti-IL-6 antibody as 50 pg mL^−1^ and the concentration of the rabbit polyclonal antibody specific for aromatase as 20 ng mL^−1^.

The aim of this work was to develop a multiple biosensor for the simultaneous determination of five biomarkers: CA125, HE4, CEA, IL-6 and aromatase, which are useful in the diagnostics of oncological and gynecological diseases. The particular components of the multiple biosensor should work for different ranges of biomarker concentrations, characteristic of such diseases. This task is more difficult than the development of a multiple biosensor working on the same concentration range. To date, such a biosensor has not been either developed or used in diagnostics.

## 2. Materials and Methods

### 2.1. Reagents

Recombinat Human CA125/MUC 16 (5609-MU) (specific activity 1.04 U µg^−1^) from Bio-techne, Warsaw, Poland, rabbit polyclonal Anti-MUC16 antibody (ab133419) from Abcam, Germany, human epididymis protein 4 (HE4) (MW of 44 kDa) (Cloud-Clone Corp., Houston, TX, USA), rabbit polyclonal antibody against HE4 (ABCAM plc, Cambridge, MA, USA), recombinant human Carcinoembryonic Antigen (CEA) (cat. no. C4835, Aldrich, Munich, Germany), mouse monoclonal anti-CEA antibody (cat. no. C2331, Aldrich, Munich, Germany), recombinant Human IL-6 (Abcam plc, Cambridge, MA, USA), mouse monoclonal anti-IL-6 antibody (Abcam plc, Cambridge, MA, USA), aromatase protein and rabbit polyclonal antibody specific for aromatase (Lucerna-Chem AG, Lucern, Switzerland), bovine serum albumin BSA (Sigma Aldrich, Munich, Germany), cysteamine hydrochloride, N-ethyl-N′-(3-dimethylaminopropyl) carbodiimide (EDC) (Sigma, Steinheim, Germany), N-Hydroxysuccinimide (NHS) (Aldrich, Munich, Germany), HBS-ES solution pH = 7.4 (0.01M HEPES, 0.15M sodium chloride, 0.005% Tween 20, 3 mM EDTA), phosphate-buffered saline (PBS) pH = 7.4, carbonate buffer pH = 8.5 (BIOMED, Lublin, Poland) photopolimer ELPEMER SD 2054 and hydrophobic protective paint SD 2368 UV SG-DG (Peters, Kempen, Germany) were used as received.

Aqueous solutions were prepared with MilliQ water (Simplicity^®^MILLIPORE, Merck KGaA, Darmstadt, Germany). The bases of the biosensors were chips covered with a layer of gold (Ssens, Enschede, The Netherlands).

The plasma samples used for the tests came from donors from the Regional Blood Donation and Blood Treatment Center in Bialystok, and from patients with ovarian cancer and endometrial cyst from the Maria Sklodowska-Curie Oncology Center. All samples were collected after obtaining the consent of the Bioethical Committee.

### 2.2. Chip and Multiple Biosensor Architecture

**Figure 1:**
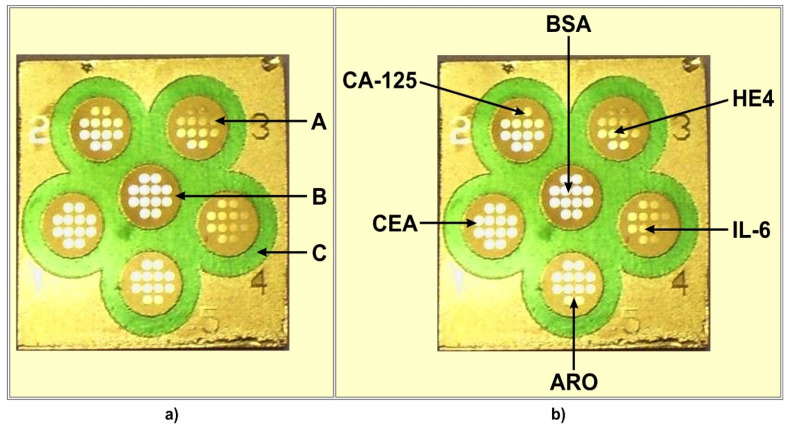
Chip (**a**) and multiple biosensor (**b**) architecture. (A) free gold surface, (B) and (C) different hydrophobic paints. (CA125), (CEA), (ARO), (IL-6), (HE4) locations of particular biosensors on the multiple biosensor. (BSA) location of BSA.

The chip used contained 6 measuring cells separated by hydrophobic paint (C) (see Figure 1a). Each cell contained 12 measuring points exposing a free gold surface (A) separated by hydrophobic paint B. The chip contains 6 × 12 measuring points with a free gold surface. 

The location of particular biosensors on the multiple biosensor is shown in Figure 1b. The sixth place contains bovine serum albumin (BSA), which is used as a reference.

### 2.3. Antibody Immobilization

The first stage of the preparation of the biosensors was the immobilisation of the linker, cysteamine. The gold chip was cleaned by rinsing with ethanol, and subsequently, water, and was dried under a stream of argon. The chip was then immersed in 20 mM ethanolic solution of cysteamine for at least 12 h. The chip with immobilized cysteamine was subsequently rinsed with ethanol and water and dried in a stream of argon. All six measuring cells (see Figure 1) were processed identically at this stage.

For the formation of a biosensor for CA125, the rabbit polyclonal anti-CA125 antibody solution (200 µg mL^−1^) in a PBS buffer (200 mM) was activated with NHS (50 mM) and EDC (200 mM) in a carbonate buffer (pH 8.5) and placed in the appropriate location on the cysteamine modified surface. For the HE 4 biosensor, the solution of rabbit polyclonal antibody against HE4 (20 ng mL^−1^) in a 200 mM PBS buffer was processed as above and placed on the corresponding measuring cell of the chip. The mouse monoclonal anti-CEA antibody at a concentration of 80 ng mL^−1^ in a 200 mM PBS buffer was treated as in the case of CA125 and placed on the measuring cell corresponding to the CEA. The mouse monoclonal anti-IL-6 antibody (50 pg mL^−1^) and rabbit polyclonal antibody specific for aromatase (20 ng mL^−1^) were treated in the same way and placed on the corresponding cells of the multiple biosensor. The BSA solution at a concentration of 1 mg mL^−1^ was placed in the cell in the centre of the multiple biosensor. The chip containing five biosensors and BSA was then incubated at 37 °C for 1 h and washed with a HBS-ES buffer and water, which were removed from the chip surface by vacuum. The multiple biosensor containing five particular biosensors was then ready for the determination of biomarker concentration. The SPRi signals were measured from the recorded images. These signals are needed for the calculation of a final result. The measurements were performed using a homemade SPRi instrument, as described previously [6].

### 2.4. Measurement of Analytical Signals

Diluted plasma samples were placed on the prepared biosensors, with 3 µL of a sample placed onto each biosensor for 10 min. The multiple biosensor was subsequently washed with an HBS-ES buffer and water, which were removed by a vacuum. Next, the second SPRi measurements were performed. Non-specific binding was monitored by measuring the SPRi signal at the sixth site of the multiple biosensor, covered with BSA. The analytical signal was calculated as the difference between the signals before and after the interaction for each measuring site and biosensor separately, and were evaluated using the calibration curve.

### 2.5. Determination of CA125, HE4, CEA, IL-6 and Aromatase by the Array SPRi Technique Using Single Biosensors

To enable a comparison of the results obtained using the multiple biosensor with those obtained using single biosensors, the concentration of CA125 was determined as described in [2], the concentration of HE4, as described in [3], the concentration of CEA as described in [4], the concentration of IL-6 as described in [5], and the concentration of aromatase as described in [6].

The biosensor for the determination of CA125 by array SPRi consists of a gold chip, cysteamine as the linker, and an immobilized rabbit polyclonal anti-CA125 antibody. The biosensor ensures the specificity of the determination, linearity of the analytical signal in the 2.2–150 µg mL^−1^ (2.9–156 U mL^−1^), an LOD of 0.66 µg mL^−1^ (0.69 U mL^−1^), and recoveries between 97 and 101% [2].

The biosensor for the determination of HE4 by the array SPRi consists of a gold chip, cysteamine as the linker and an immobilized rabbit polyclonal antibody against HE4. The biosensor ensures the specificity of the determination, linearity of the analytical signal in the range 2–120 pM (0.088–5.28 ng mL^−1^), an LOD of 0.088 ng mL^−1^, and recoveries between 102 and 103.5% [3].

The biosensor for the determination of CEA by array SPRi consists of a gold chip, cysteamine as the linker and an immobilized mouse monoclonal anti-CEA antibody. The biosensor ensures the specificity of the determination, linearity of the analytical signal in the range 0.40–20 ng mL^−1^, an LOD of 0.12 ng mL^−1^, and recoveries between 101% and 104% [4].

The biosensor for the determination of IL-6 by array SPRi consists of a gold chip, cysteamine as the linker and an immobilized mouse monoclonal anti-IL-6 antibody. The biosensor ensures the specificity of the determination, linearity of the analytical signal in the range 3–20 pg mL^−1^ with LOQ of 3 ng mL^−1^ and recoveries between 101 and 105% [5].

The biosensor for the determination of aromatase by array SPRi consists of a gold chip, cysteamine as the linker and an immobilized rabbit polyclonal antibody specific for aromatase. The biosensor ensures the specificity of the determination, linearity of the analytical signal in the range 0.3–5 ng mL^−1^, an LOQ of 0.3 ng mL^−1^ and recoveries between 97 and 108% [6].

## 3. Results

### 3.1. Calibration of the Multiple-Array Biosensor

Prior to the application of the multiple-array biosensor for the measurement of biomarker concentrations in real samples, the calibration of the multiple biosensor was performed. This was achieved within the ranges indicated in Table 1. Six concentrations of a particular biomarker were used for the construction of a calibration curve for that biomarker. The measurements were repeated five times for each concentration. The calculated calibration curves are shown in Table 1.

### 3.2. Comparison of Results from the Multiple Biosensor with Those Obtained Using Single Biosensors

**Figure 2:**
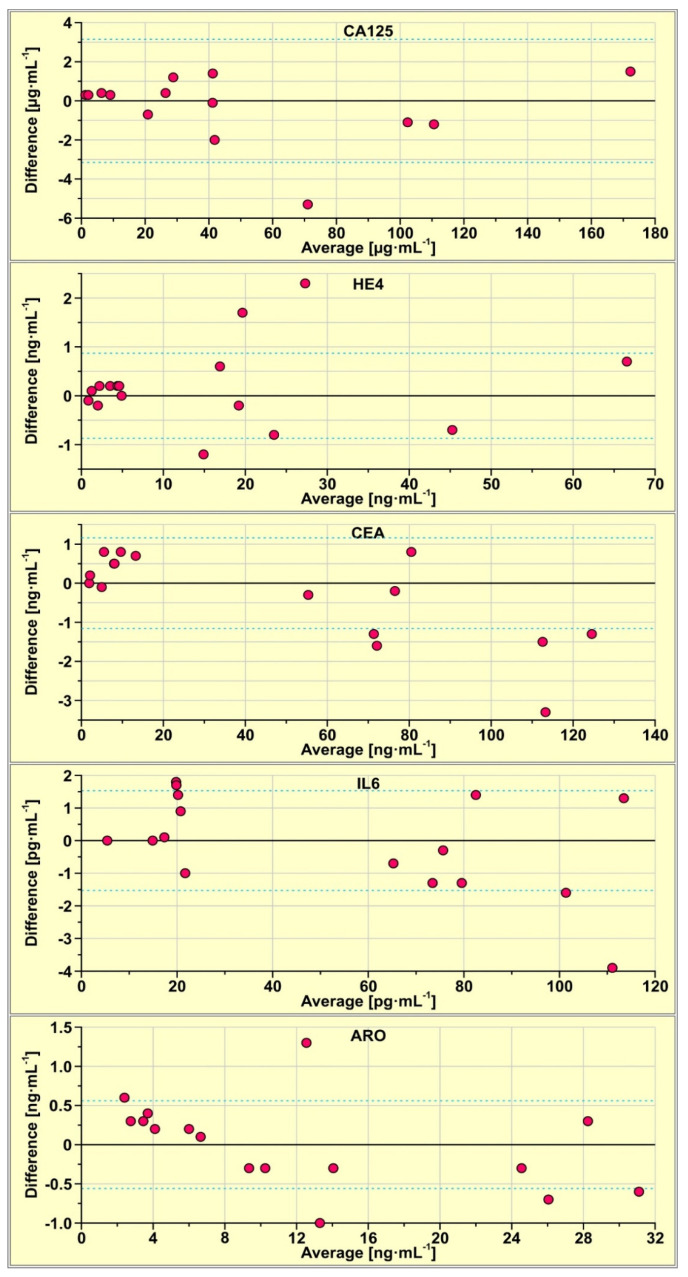
Bland-Altman plots of the results obtained with the multiple biosensor and those obtained with single biosensors.

Concentrations of CA125, HE4, CEA, IL-6 and aromatase in 15 real samples of serum were determined using the multiple biosensor and simultaneously using the single biosensors. The results are compared using Bland–Altman plots. This tool is used for the identification of potential differences between the results from two methods, that is, those obtained with the multiple biosensor and those obtained with single biosensors, used in separate experiments. For each sample, the difference between the result obtained with the multiple biosensor and that obtained with a single biosensor is calculated. This difference (Y-axis) is plotted against the average of those results (X-axis). The results are shown in Figure 2, for each biosensor separately. Dashed lines show the values of the SD of the difference. The majority of the results are located within the ±SD range, showing that the results obtained with the multiple biosensor are in principle the same as those obtained with the single biosensors, the degree of this agreement is different for various biosensors. In the case of the biosensor for the CA125 determination, only one of the 14 results is located above the SD, while in the cases of CEA and aromatase, three out of 15 results lie beyond the SD. 

### 3.3. Examples of CA125, HE4, CEA, Il-6 and Aromatase Determination with the Multiple Biosensor in Real-Blood Serum Samples

**Figure 3:**
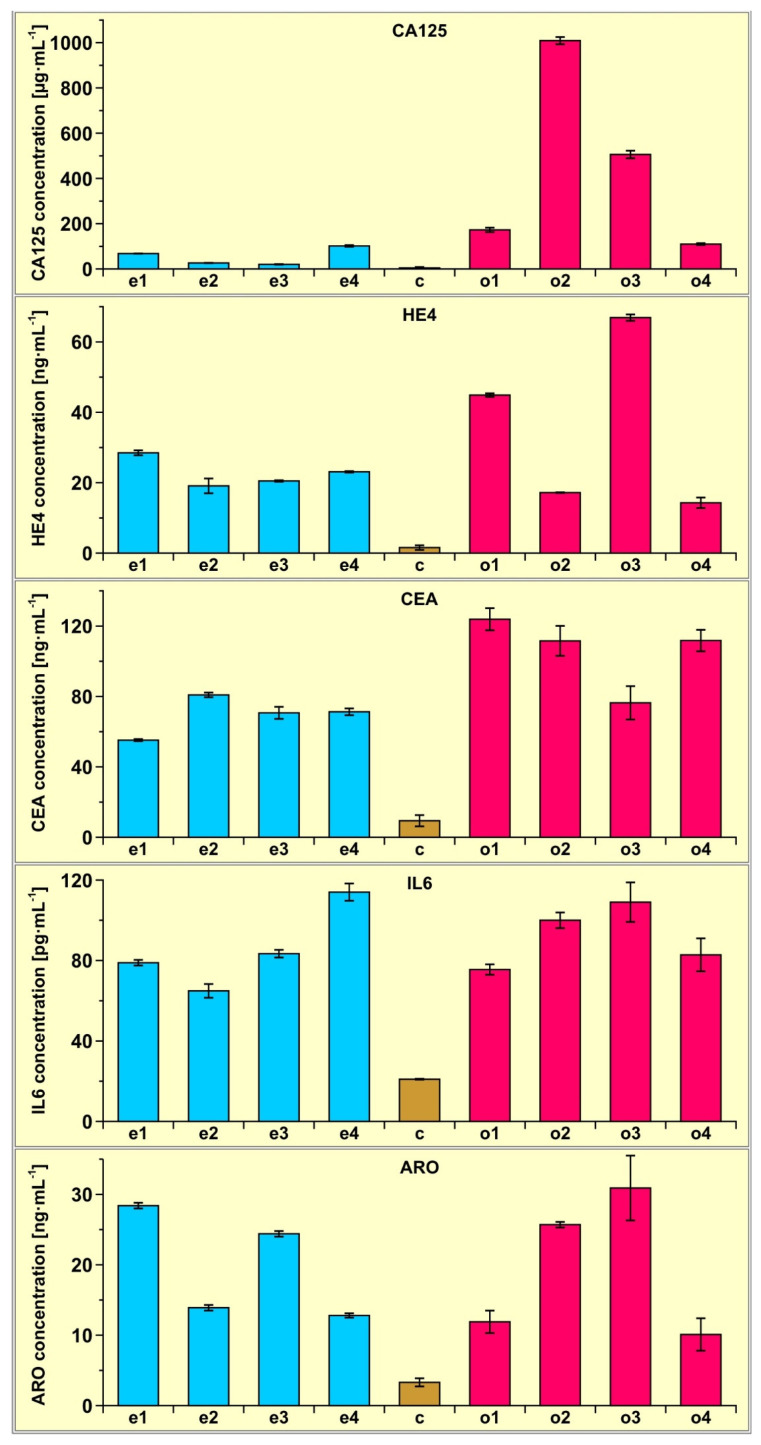
Results of CA125, HE4, CEA, Il-6 and aromatase determination in the plasma of four patients with endometrial cysts (samples e1, e2, e3 and e4) and four patients with ovarian cancer (samples o1, o2, o3 and o4) determined using the multiple biosensor. The average of four control samples (c) is also included.

To demonstrate the potential of the multiple biosensor, four samples of plasma from patients with an endometrial cyst and four samples of plasma from patients with ovarian cancer were determined using the multiple biosensor. Four samples of plasma from healthy subjects were also determined for comparison. Each measurement was repeated five times. The results are shown in Figure 3.

The experiments performed enable an analysis of the precision of determination of the biomarkers by multiple-array SPRi. The results of this analysis are shown in Table 2.

The average precision is quite good, taking into account the complexity of blood serum as a matrix and the generally low level of determined concentrations (pg mL^−1^ for IL-6, ng mL^−1^ for HE4, CEA and aromatase and µg mL^−1^ for CA-125). Ranges of precision for particular samples generally follow the rule that the lower the level of concentration, the worse the precision. However, the precision of aromatase determination is poorer than expected, based on the level of that biomarker, and reveals a need for work to improve this parameter.

## 4. Discussion

The results obtained show that the simultaneous determination of CA125, HE4, CEA, IL-6 and aromatase with a multiple-array SPRi biosensor is feasible. The results are in principle the same as those obtained with the corresponding single biosensors, as is shown by the Bland–Altman plots in Figure 2. Moreover, the multiple biosensor performs works well despite the large difference in the levels of particular biomarkers in blood plasma, which range from pg mL^−1^ for the determination of IL-6, ng mL^−1^ for HE4, and CEA and aromatase to µg mL^−1^ for CA125. A significant factor that enables the construction of the multiple biosensor is the simple structure of the individual biosensors. They consist only of a linker and a covalently attached antibody. This is an advantage of the array SPRi technique over other SPR or SPRi techniques. For example, the array SPRi biosensor for the determination of CEA consists of cysteamine liker and covalently bonded anti-CEA antibody, while a fluidic SPR system biosensor requires, in order to attain necessary sensitivity, the use of a primary anti-CEA antibody and a secondary anti-CEA antibody conjugated with gold nanoparticles [43,44]. The measuring of the CEA concentration by array SPRi consists of a single step, while the measuring process in the fluidic SPR consists of two steps: the entrapment of CEA and treatment with the secondary antibody conjugated with gold nanoparticles. Such a measuring process is hardly feasible when several different biosensors are used, each of them being processed in a different way. Thus, the array SPRi technique has the potential to enable the creation of multiple biosensors. There are interesting alternatives to the simple covalent immobilisation of an antibody: immobilization of the antibody via cross-linked Protein G [45], or the use of a fragmented antibody instead of the ‘normal’ antibody [46]. 

The developed multiple biosensor is a new tool offering benefits to oncologists and gynecologists. It may improve liquid biopsy, namely diagnosis based on the determination of biomarkers in blood plasma/serum or other body fluids. The simultaneous determination of several biomarkers may be an excellent tool for the screening of the population for earlier detection of diseases. The choice of CA125, HE4, CEA, IL-6 and aromatase was based on the availability of single biosensors for these biomarkers. The values of CA-125 and HE 4 concentrations determined with the multiple biosensor can be used for calculations in the ROMA algorithm (risk of ovarian malignancy algorithm) [47]. This calculation is different for premenopausal patients and postmenopausal patients. In both cases the algorithm is determined by the formula:

ROMA = exp (PI)/[1 + exp (PI)] × 100%(1)

where PI is a predictive index calculated for premenopausal patients

PI = 2.38 × ln(HE4) + 0.0626 × ln(CA125) − 12.0(2)

with a cut-off value 11.4%, and for postmenopausal patients

PI = 1.04 × ln(HE4) + 0.732 × ln(CA125) − 8.09(3)

with a cut-off value 29.9%.

It is highly possible that another set of biosensors would be better suited to diagnostic purposes. The authors wait critical comments and suggestions concerning the selection of particular biosensors to constitute the multiple biosensor. Recently, a modification of the ROMA algorithm was proposed, which included the third biomarker thymidine kinase (TK1) [48]. This new algorithm is called ROMI (risk of ovarian malignancy index). Moreover, it seems possible to create a multiple biosensor having a number of biosensors greater than five; for example, nine. The flexibility of the array SPRi technique in this regard is one of its advantages.

Certainly, these several examples do not provide a sufficient basis to draw conclusions of a medical nature. However, even such a small number of samples shows that the concentration of the investigated biomarkers is significantly higher in the case of ovarian cancer and endometrial cyst than in the controls, and that CA125 determination in plasma can be used to distinguish between ovarian cancer and endometrial cyst.

## 5. Conclusions

Diagnostics based on the determination of biomarkers in body fluids will be more successful when several biomarkers are determined. A multiple-array SPRi biosensor has been developed for the simultaneous determination of CA125, HE4, CEA, IL-6 and aromatase, which are useful in the diagnostics of oncological and gynecological diseases. The particular components of the multiple biosensor work in different concentration ranges, characteristic for such diseases. The results obtained with the multiple-array biosensor are very similar to those obtained with a single biosensor. The average precision of determination is quite good. To date, no such biosensor has been developed or used in diagnostics.

The applicability of the multiple biosensor was demonstrated using several examples of plasma from patients suffering from ovarian cancer and endometrial cyst.

It seems possible to create a multiple biosensor having a number of biosensors greater than five and another set of biosensors that would be suited to various diagnostic purposes.

## Figures and Tables

**Table 1:** Calibration curves and concentration ranges for particular biosensors included in the multiple-array biosensor.

Biomarker	Calibration Curve	Range/Units	R^2^
CA125	Y = 41.13X + 146	2.2–150/µg mL^−1^	0.995
HE4	Y = 557.3X + 227	0.088–5.28/ng mL^−1^	0.991
CEA	Y = 151.5X + 85	0.40–20/ng mL^−1^	0.993
IL-6	Y = 126X + 7.4	3–20/pg mL^−1^	0.992
Aromatase	Y = 1408X + 145	0.3–5/ng mL^−1^	0.994

X—biomarker concentration; Y—arbitrary units.

**Table 2:** Precision of measurements of biomarker concentration in eight real samples.

Biomarker	RSD [%]	Average [%]	Range [%]
CA125	0.73; 3.0; 5.8; 3.5; 5.5; 1.6; 3.3; 3.8	3.4	0.73–5.8
HE4	2.5; 11; 1.0; 0.86; 1.1.; 0.58; 1.3; 10	3.5	0.58–11
CEA	1.1; 1.6; 4.8; 2.7; 5.1; 7.6; 12; 5.4	5.0	1.1–12
IL-6	1.8; 5.2; 2.3; 3.8; 3.4; 3.9; 9.0; 11	5.0	1.8–11
Aromatase	1.4; 2.9; 1.6; 2.3; 13; 1.6; 15; 23	7.6	1.4–23

## Data Availability

Not applicable.
